# Theoretical Study on Photocatalytic Reduction of CO_2_ on Anatase/Rutile Mixed-Phase TiO_2_

**DOI:** 10.3390/molecules29174105

**Published:** 2024-08-29

**Authors:** Jieqiong Li, Shiyu Wei, Ying Dong, Yongya Zhang, Li Wang

**Affiliations:** 1 Henan Engineering Center of New Energy Battery Materials, College of Chemistry and Chemical Engineering, Shangqiu Normal University, Shangqiu 476000, China; lijqchem@foxmail.com (J.L.); weishiyu1015@126.com (S.W.); dongying0928@126.com (Y.D.); 2 Henan Key Laboratory of Protection and Safety Energy Storage of Light Metal Materials, Henan University, Kaifeng 475004, China

**Keywords:** CO_2_ photoreduction, mixed-phase TiO_2_, band alignment, charge transport, density functional theory

## Abstract

The construction of anatase/rutile heterojunctions in TiO_2_ is an effective way of improving the CO_2_ photoreduction activity. Yet, the origin of the superior photocatalytic performance is still unclear. To solve this issue, the band edges between anatase and rutile phases were theoretically determined based on the three-phase atomic model of (112)_A_/II/(101)_R_, and simultaneously the CO_2_ reduction processes were meticulously investigated. Our calculations show that photogenerated holes can move readily from anatase to rutile via the thin intermediated II phase, while photoelectrons flowing in the opposite direction may be impeded due to the electron trapping sites at the II phase. However, the large potential drop across the anatase/rutile interface and the strong built-in electric field can provide an effective driving force for photoelectrons’ migration to anatase. In addition, the II phase can better enhance the solar light utilization of (112)_A_/(100)_II_, including a wide light response range and an intensive optical absorption coefficient. Meanwhile, the mixed-phase TiO_2_ possesses negligible hydrogenation energy (CO_2_ to COOH*) and lower rate-limiting energy (HCOOH* to HCO*), which greatly facilitate CH_3_OH generation. The efficient charge separation, strengthened light absorption, and facile CO_2_ reduction successfully demonstrate that the anatase/rutile mixed-phase TiO_2_ is an efficient photocatalyst utilized for CO_2_ conversion.

## 1. Introduction

The photocatalytic reduction of CO_2_ into high-value-added chemicals and fuels on semiconductors is an efficient and clean energy conversion technology which has been considered to be a promising approach to simultaneously solving energy and environmental issues [1,2,3]. Among the various semiconductor materials, TiO_2_ is usually taken as a prototypical photocatalyst for CO_2_ conversion owing to its merits including environmentally friendliness, low cost, and long-term photochemical stability [4,5,6]. At present, enormous efforts have been devoted to enhance the photocatalytic activity of CO_2_ reduction on TiO_2_ [7]. For instance, Liu et al. [8] have pointed out that introducing oxygen vacancies, which can act as active sites, is beneficial to CO_2_ binding, activation, and dissociation. In addition, Umezawa et al. [9] have demonstrated that loading Pt clusters on TiO_2_’s surface can promote the photocatalytic performance of CO_2_ reduction by facilitating the ionization of HCOO^-^ and OH^-^ on the surface. In addition, fabrication of heterogeneous semiconductors is also regarded as an effective strategy to increase the photocatalytic activity due to the efficient charge separation and migration across the junction interface, such as the design of anatase (100)/(001) surface heterojunctions [10] and TiO_2_/ZnIn_2_S_4_ phase heterojunctions [11]. Alternatively, anatase/rutile mixed-phase TiO_2_ has been demonstrated to be a good photocatalyst with remarkable photocatalytic CO_2_ reduction capabilities due to the synergistic effect between anatase and rutile phases [12,13,14].

Although the anatase/rutile mixed-phase TiO_2_ as a photocatalyst for CO_2_ reduction has been extensively studied, a profound understanding of the detailed reaction pathways from CO_2_ to the reduced products is lacking, both experimentally and theoretically. Consequently, the origin of the superior photocatalytic performance on mixed-phase TiO_2_ is still obscure. This is largely attributed to the fact that the fundamental mechanism of charge separation and transport, i.e., how the photogenerated electrons and holes transfer across the anatase/rutile phase junctions, is still in dispute. Some hold the view that the photogenerated electrons preferentially move from anatase to rutile [15,16]. Meanwhile, Li et al. found a built-in electric field across the anatase/rutile interface with a upward band bending from anatase to rutile, which can facilitate the photogenerated electrons’ transport from rutile to anatase [17]. These inconsistent results cause the crystal phase for the CO_2_ reduction reaction being confusing. Generally, the charge migration direction across the interface is closely related to the band alignment between phases. Therefore, clarifying the band edge positions of anatase and rutile in mixed-phase TiO_2_ is helpful to understanding the charge migration mechanism. Theoretical simulations can provide valuable insights into the electronic band structures of photocatalysts at the atomic level. However, because no atom-resolved anatase/rutile phase junctions have been reported experimentally, constructing a realistic structural model for anatase/rutile TiO_2_ heterophase junctions is a challenging task.

At present, the commonly used theoretical models of the anatase/rutile interface are direct interface models with a two-phase junction, i.e., direct contact between anatase and rutile atomic surfaces. For instance, Deskins et al. established the anatase/rutile interface by connecting the anatase (101) with rutile (110) [18], which are the thermodynamically most stable surfaces for anatase and rutile, respectively. However, the combination of (101)_A_/(110)_R_ may generate a large interfacial strain and induce a disordered interfacial structure, which is unfavorable for the transport of photogenerated charge carriers at the interface. Instead, some researchers have proposed constructing interfacial models using TiO_2_ surfaces with relatively high energies. These researchers believed that the highly active surfaces may have a stronger tendency to form the heterojunction interfaces than the stable surfaces. However, a variety of combinations have been reported, including (001)_A_/(101)_R_ [19], (100)_A_/(100)_R_ [20] and (101)_A_/(111)_R_ [21]. Later, Liu et al. built an indirect interface model for anatase/rutile with a structurally ordered three-phase junction, i.e., involving an intermediate phase (TiO_2_-II (α-PbO_2_-like form)) between anatase and rutile ((112)_A_/II/(101)_R_) [22]. The intermediate II phase, despite having thin atomic layers, is essential to alleviate the interfacial strain energy and stabilize the anatase/rutile interface, thus promoting the electron and hole migration and separation and enhancing the photoactivity. Importantly, this indirect interface model is consistent with the macroscopic structure of anatase/rutile crystals synthesized by Hosono et al. [23]. Although Liu et al. have predicted the charge migration direction across the anatase/rutile heterophase junction, the transport mechanism is a rough estimation, which is obtained on the basis of the computed energetics of charge carriers and the experimental band gaps for anatase and rutile.

Hence, to unravel the mechanism of charge separation and transport across the anatase/rutile interface and explore the influence of the phase junction on the electron–hole migration, in this study we presented an ab initio calculation on the band edge positions of the indirect three-phase junction model of (112)_A_/II/(101)_R_. In addition, the detailed mechanistic pathways of CO_2_ photoreduction on the anatase/rutile mixed-phase TiO_2_ were meticulously investigated to propose an optimal reduction reaction route. It is anticipated that this work can help us to deeply understand the role of phase junctions in semiconductor photocatalysis and provide a theoretical guidance for experimental researchers to design efficient TiO_2_-based heterostructures.

## 2. Results and Discussion

### 2.1. Structural Modeling

To clarify the charge migration mechanism in anatase/rutile mixed-phase TiO_2_, a reasonable structural model of the heterophase junction is initially required. According to Liu et al.’s work [22], the three-phase junction model of (112)_A_/II/(101)_R_ is a nanopin-like structure, i.e., an aperiodic system. Due to the limitations of periodic boundary conditions, this indirect model could not be constructed directly. Therefore, we divided the entire three-phase model ((112)_A_/II/(101)_R_) into two separate biphase models, namely (112)_A_/II and II/(101)_R_. The indirect three-phase junction model can be obtained by rotating the II/(101)_R_ junction by 90° with respect to the (112)_A_/II junction and then attaching it to the (112)_A_/II structure via the intermediate II phase. Based on the structural information from SSW sampling pathways [24,25,26], the intermediate phases in the (112)_A_/II and II/(101)_R_ models correspond to two different crystal facets, namely the (100) surface (i.e., (112)_A_/(100)_II_) and the (001) surface (i.e., (001)_II_/(101)_R_).

Prior to the contact, the lattice parameters of anatase (112) and II (100) in the *xy* dimensions are *a* = 5.44 Å, *b* = 5.34 Å and *a* = 4.93 Å, *b* = 5.60 Å, respectively (*a* = 4.63 Å, *b* = 5.45 Å for rutile (101) and *a* = 4.59 Å, *b* = 5.60 Å for II (001)). It can be seen that there is a lattice mismatch between anatase (or rutile) and the intermediate II phases, especially between anatase (112) and II (100) (9.4% and 4.9% along *a* and *b* directions, respectively). This large lattice mismatch (>3.0%) will lead to a significant strain and a poor Ti–O match at the interface. Liu et al. suggested that the lattice-misfit strain can be released by reducing the number of atomic layers in the intermediate II phase [22], contributing to a good cation–anion match in the contact region. Therefore, we assume that the lattice parameters of II (100) and II (001) in the biphase models are determined by anatase and rutile phases, respectively, which are consistent with those of anatase (112) and rutile (101). Meanwhile, because II (001) attaches to II (100) by rotating 90°, the lattice of II (001) in the *c* direction corresponds to that of II (100) in the *a* direction. Similarly for II (100), its lattice parameter in the *c* axis is equal to that of II (001) in the *a* axis.

**Figure 1:**
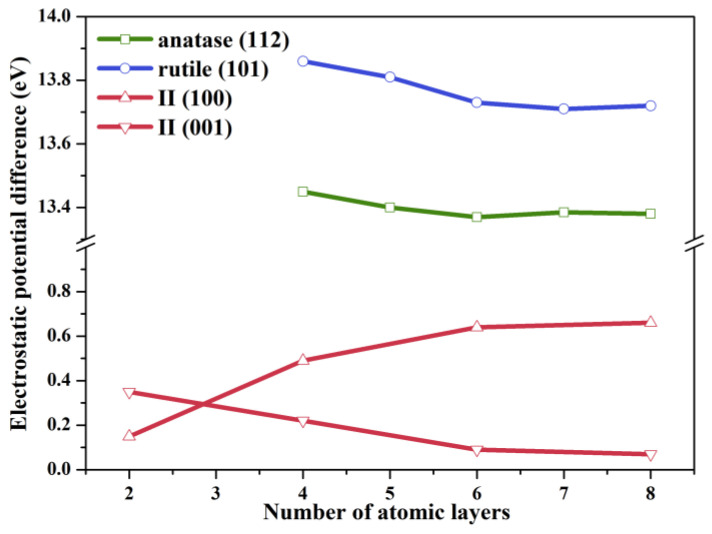
The average electrostatic potential difference plotted against the TiO_2_ slab thickness of anatase (112), rutile (101), II (100), and II (001) surfaces.

**Figure 2:**
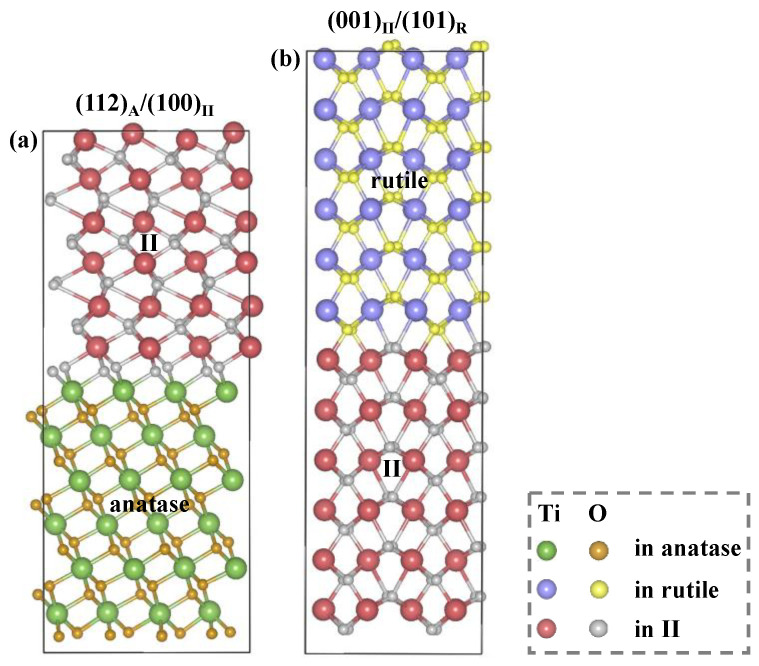
Optimized geometric heterostructures of (**a**) (112)_A_/(100)_II_ and (**b**) (001)_II_/(101)_R_. The “II” stands for the intermediate phase (TiO_2_-II).

In addition to the lattice parameters, another important aspect to be taken into account in the construction of interfacial structures is the thickness of TiO_2_ slabs. We first studied the pure-phase TiO_2_ of anatase (112) and rutile (101) in vacuum, which were both modeled with 2 × 2 supercells in the *xy* dimensions. Then, the electrostatic potentials of these two TiO_2_ surfaces with different atomic layers were calculated (see Tables S1 and S2). By analyzing the convergence of electrostatic potential difference (∆V, the difference in average electrostatic potentials between TiO_2_ and vacuum phases) with the slab thickness (see Figure 1), it is found that the ∆V of both anatase (112) and rutile (101) displays fast convergence at six atomic layers with small oscillations not exceeding 0.02 eV from six- to eight-layer slabs. Therefore, we deemed that the appropriate TiO_2_ slabs for anatase (112) and rutile (101) are both six atomic layers thick. Regarding the intermediate phases of II (100) and II (001), we evaluated their atomic thickness using the biphase junction models of (112)_A_/(100)_II_ and (001)_II_/(101)_R_. Based on the computed electrostatic potentials (see Tables S3 and S4) and the dependence of ∆V (the difference in average electrostatic potentials between anatase (or rutile) and intermediate II phases) on slab thickness (see Figure 1), a six-layer slab was also chosen for both II (100) and II (001) to ensure the convergence of the TiO_2_ slab. Following the above analysis, the reasonable heterojunction models of (112)_A_/(100)_II_ and (001)_II_/(101)_R_ were established, as illustrated in Figure 2.

### 2.2. Electronic Properties

**Figure 3:**
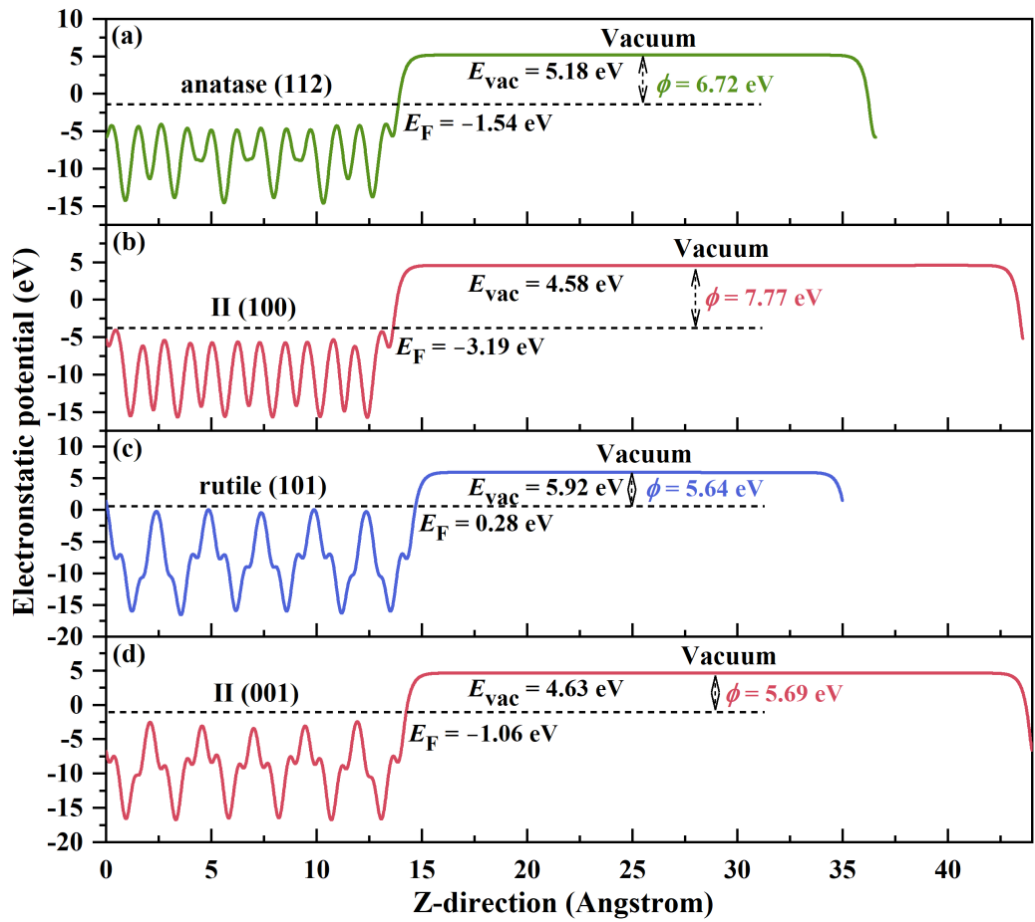
Calculated work functions of an (**a**) anatase (112) surface, (**b**) II (100) surface, (**c**) rutile (101) surface, and (**d**) II (001) surface.

Due to the disequilibrium in the electrochemical potentials of electrons, when two semiconductors are in contact, charge transfer will occur at the interface until a unified Fermi level is reached. Work function (ϕ) is a key quantity for determining the behavior of charge transfer and can be defined as [27]

(1) $$\phi =E_{vac}-E_{F}$$

where $E_{vac}$ and $E_{F}$ are the vacuum level and Fermi level, respectively. Figure 3 displays the profiles of electrostatic potential along the Z direction normal to the TiO_2_ surfaces. As shown in Figure 3a,b, the work function of II (100) is 7.77 eV, which is higher than that of anatase (112), with 6.72 eV. This indicates that electrons will transfer from anatase (112) to II (100) until the Fermi levels achieve equilibrium. Meanwhile, rutile (101) and II (001) have approximately equivalent work functions (5.64 eV for rutile (101) and 5.69 eV for II (001), see Figure 3c,d). The comparable ϕ values will provide a weak driving force for electron transfer.

**Figure 4:**
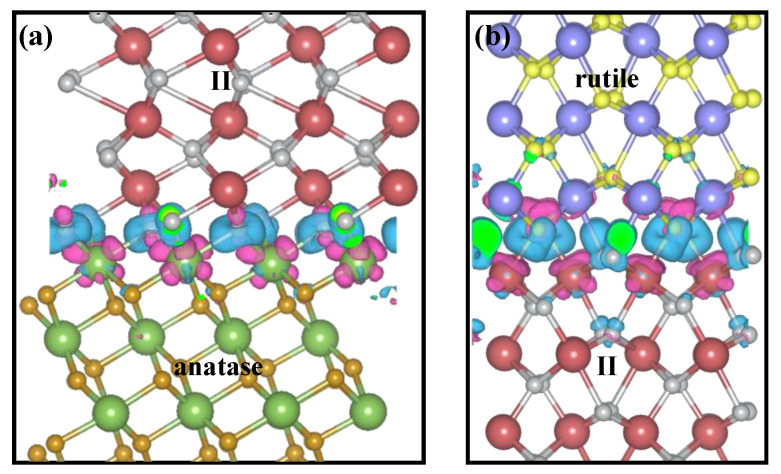
Planar-averaged electron density difference in the Z direction for (**a**) (112)_A_/(100)_II_ and (**b**) (001)_II_/(101)_R_ heterostructures. The pink and blue isosurfaces represent the electron depletion and accumulation, respectively. The “II” stands for the intermediate phase (TiO_2_-II).

To gain a clear understanding of the charge transfer process at the phase junction, the charge density difference ($∆\rho_{e}$) was further calculated as follows:

(2) $$∆\rho_{e}=\rho_{e}\left(i/ii\right)-\rho_{e}\left(i\right)-\rho_{e}(ii)$$

where $\rho_{e}\left(i/ii\right)$, $\rho_{e}\left(i\right)$, and $\rho_{e}(ii)$ are the electron densities of the i/ii biphase junction, pure phase i, and pure phase ii, respectively. The three-dimensional charge density difference maps for (112)_A_/(100)_II_ and (001)_II_/(101)_R_ are plotted in Figure 4. The pink- and blue-colored isosurfaces represent the charge depletion and accumulation regions, respectively. It can clearly be seen that the main charge density changes are localized at the interface. For (112)_A_/(100)_II_, the pink isosurfaces appear around the Ti atoms in the anatase phase, while the blue isosurfaces distribute in the O atoms in the intermediate II phase (see Figure 4a). This suggests that there is a charge transfer from anatase (112) to II (100), which is in accordance with the results for the work functions. In the case of (001)_II_/(101)_R_, although the charge transfer also occurs between Ti and O atoms, it happens within the respective phase of rutile (101) and II (001) rather than between the two phases (see Figure 4b). This observation provides direct evidence for the aforementioned conclusion that there is less charge transfer across the (001)_II_/(101)_R_ interface when they are in contact.

The strong charge transfer can cause a distinct charge redistribution at the (112)_A_/(100)_II_ interface, leading to the creation of an internal built-in electric field and an upward band bending from anatase (112) to II (100) across the phase junction. Upon light illumination, the built-in electric field can provide a direct driving force for the photoelectrons’ transfer from II (100) to anatase (112). However, almost no charge transfer between rutile (101) and II (001) may induce a weak built-in electric field and a negligible band bending. These deductions offer a good explanation for the experimental finding that to achieve good photocatalytic performance for water oxidation, the concentration of the anatase phase in an anatase/rutile heterostructure catalyst should be much higher than that of rutile, i.e., anatase (80%)/rutile (20%) [28,29]. The abundance of the anatase phase is required to form a high concentration of the (112)_A_/(100)_II_ interface, which is responsible for creating a strong built-in electric field, thereby accelerating the separation and transport of photogenerated charge carriers.

### 2.3. Charge Migration Mechanism

**Figure 5:**
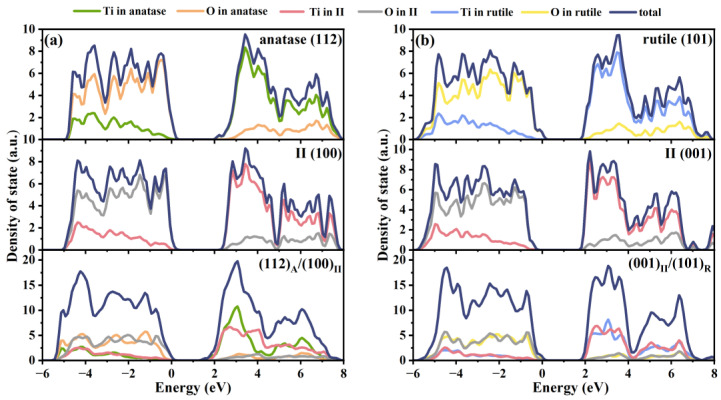
Projected density of states (PDOS) of (**a**) (112)_A_/(100)_II_ before and after contact and (**b**) (001)_II_/(101)_R_ before and after contact. The Fermi level is set at 0 eV.

**Figure 6:**
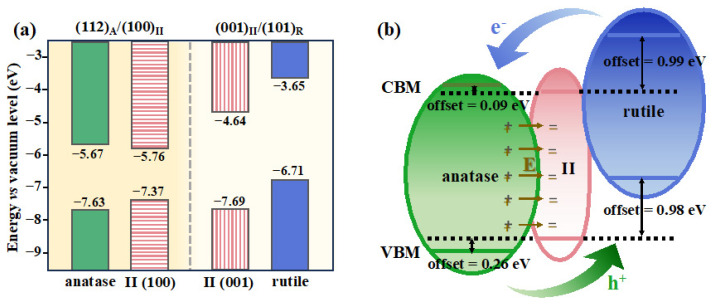
(**a**) Band edge positions relative to the vacuum level of (112)_A_/(100)_II_ and (001)_II_/(101)_R_ heterostructures. (**b**) Illustrations of the proposed band alignment and the charge transfer mechanism in anatase/rutile mixed-phase TiO_2_. The “II” stands for the intermediate phase (TiO_2_-II).

Based on the optimized biphase junction models, the projected density of states (PDOS) and the band edge positions of (112)_A_/(100)_II_ and (001)_II_/(101)_R_ with respect to the vacuum level were calculated using the hybrid HSE06 functional, as shown in Figure 5 and Figure 6, respectively. It is noteworthy that for both pure-phase TiO_2_ (anatase (112), II (100), II (001), and rutile (101)) and mixed-phase TiO_2_ ((112)_A_/(100)_II_ and (001)_II_/(101)_R_), the CBM positions are primarily derived from the Ti-3*d* orbitals, while the VBM positions are mainly contributed by the O-2*p* orbitals. Additionally, the formation of (112)_A_/(100)_II_ heterojunctions moves the CBM to a lower-energy position, resulting in an obvious reduction in the band gap compared to those of anatase (112) and II (100) (see Figure 5a). Meanwhile, the band gap of the (001)_II_/(101)_R_ heterojunction remains almost unchanged (see Figure 5b). In Figure 6a, it can be seen that for (112)_A_/(100)_II_, the valence and conduction band edges of anatase (112) straddle those of II (100), which is unfavorable for the separation of electron–hole pairs. For (001)_II_/(101)_R_, the VBM positions of rutile (101) and II (001) are −6.71 and −7.69 eV, respectively, while the CBM positions are −3.65 and −4.64 eV, respectively. Both the VBM and CBM positions of rutile (101) are obviously higher than those of II (001), leading to a staggered type II heterojunction. Accordingly, the (001)_II_/(101)_R_ interface is beneficial for electron and hole accumulation on II (001) and rutile (101), respectively. The relative band levels are in agreement with Liu et al.’s results obtained by calculating the energy difference for the electrons and holes [22].

**Figure 7:**
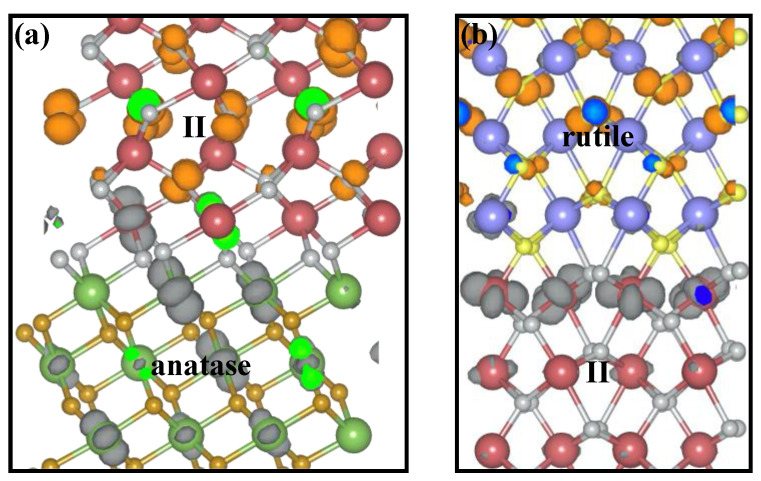
Spatial representations of electron and hole distributions of (**a**) (112)_A_/(100)_II_ and (**b**) (001)_II_/(101)_R_ heterostructures. Gray and orange areas represent the electron and hole distributions, respectively. The “II” stands for the intermediate phase (TiO_2_-II).

Arranging the band edge positions of (112)_A_/(100)_II_ and (001)_II_/(101)_R_ together, we can summarize the overall band alignment of the indirect three-phase junction in anatase/rutile mixed-phase TiO_2_ (see Figure 6b). This figure shows that the VBM positions of the three phases decrease following the order of rutile > II > anatase. The VBM of rutile is much higher than that of anatase, by 1.24 eV. Consequently, the photogenerated holes can transfer easily from anatase to rutile via the intermediate II phase without a barrier. By contrast, due to the slightly higher conduction band edge of anatase than II, after the photogenerated electrons flow from rutile to II, the electrons may be trapped at the intermediate phase. However, the CBO between the anatase and II phases is only 0.09 eV. Taking into account the computational errors, such a small energy difference can almost be ignored. In addition, the thickness of the intermediate II phase is relatively thin. Thus, the trapped electrons can readily reach saturation and then be driven to the anatase side by the large potential drop between the anatase and rutile phases (0.90 eV). Furthermore, as mentioned above, there is a built-in electric field at the (112)_A_/(100)_II_ interface directed from anatase (112) to II (100), which is conducive to the photogenerated electrons’ transfer from intermediate II to the anatase phase. To give an intuitive picture about the charge migration direction upon light excitation, the spatial distributions of electrons and holes (gray and orange, respectively) corresponding to the strongest absorptions of (112)_A_/(100)_II_ (580 nm) and (001)_II_/(101)_R_ (614 nm) are depicted in Figure 7, which originate from the S_0_ → S_40_ and S_0_ → S_41_ excitations, respectively. The electron–hole analysis was performed using the Multiwfn software package (version 3.7) [30]. It can clearly be seen that for (112)_A_/(100)_II_, the electrons and holes generated upon photoexcitation are mainly localized at the anatase and intermediate II phases, respectively, while for (001)_II_/(101)_R_, the photogenerated electrons and holes are distributed in the intermediate II and rutile phases, respectively. Driven by the combined effect of various factors, it can be concluded that in anatase/rutile mixed-phase TiO_2_, the three-phase junction can prompt the photogenerated electrons to flow to the anatase phase while the holes transfer to the rutile phase, leading to an efficient spatial separation of electron–hole pairs. This charge migration mechanism is consistent with the observation by Li et al. [31]. Experimentally, they showed that the mixed-phase TiO_2_ exhibiting superior photoelectrochemical water splitting is the TiO_2_–AR electrode with rutile as the external layer, which possesses an appropriate phase alignment for forward electron migration from rutile to anatase.

**Figure 8:**
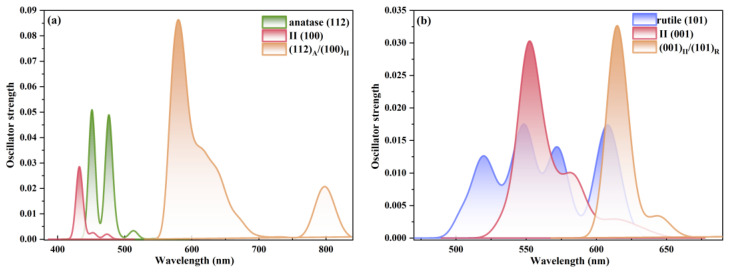
Simulated optical absorption spectra of (**a**) (112)_A_/(100)_II_ before and after contact and (**b**) (001)_II_/(101)_R_ before and after contact.

In addition to the efficient charge separation and transfer, high-performance semiconductor photocatalysts also require effective solar light utilization. Therefore, the optical absorption spectra of biphase TiO_2_ ((112)_A_/(100)_II_ and (001)_II_/(101)_R_) were investigated, as illustrated in Figure 8. For comparison, the optical absorption properties for pure-phase TiO_2_ (anatase (112), II (100), II (001) and rutile (101)) were also included. One can see that the maximum absorption peaks for pure anatase (112) and II (100) are both located at about 500 nm. After the formation of heterojunctions, the (112)_A_/(100)_II_ heterostructure shows an obvious redshift (about 800 nm) and possesses a larger optical absorption coefficient (see Figure 8a). The redshift is attributed to its reduced band gap (see Figure 5a). The broad and strong optical absorption can promote the photoexcitation of electrons and holes. For (001)_II_/(101)_R_, the maximum absorption peak slightly shifts to the red light region and the optical absorption coefficient mildly increases in comparison with those of rutile (101) and II (001) (see Figure 8b). The above findings indicate that the interfacial interaction between anatase (112) and II (100) plays a more significant role in the light absorption than that between rutile (101) and II (001). In addition, in anatase/rutile mixed-phase TiO_2_, anatase phase should act as a photon adsorption agent, which is in accordance with previous experimental and theoretical studies [22,32].

### 2.4. CO_2_ Photoreduction Mechanism

Driven by the spatial charge separation, the photogenerated holes and electrons will preferentially accumulate on rutile and anatase phases, respectively. The photogenerated electrons in the conduction band of anatase can trigger CO_2_ conversion. Therefore, the CO_2_ reduction reaction prefers to proceed on the anatase phase. Moreover, (112)_A_/(100)_II_ heterostructure possesses superior light absorption ability, which is conductive to enhancing its photocatalytic activity. Therefore, the anatase/rutile mixed-phase TiO_2_ is regarded as a preferable photocatalyst for CO_2_ conversion. Next, the CO_2_ reaction processes on the anatase side of (112)_A_/(100)_II_ heterostructure were systematically explored. In order to reveal the influence of heterojunctions on CO_2_ reduction, we also took into account the reaction pathways on pure-phase anatase for comparison.

**Figure 9:**
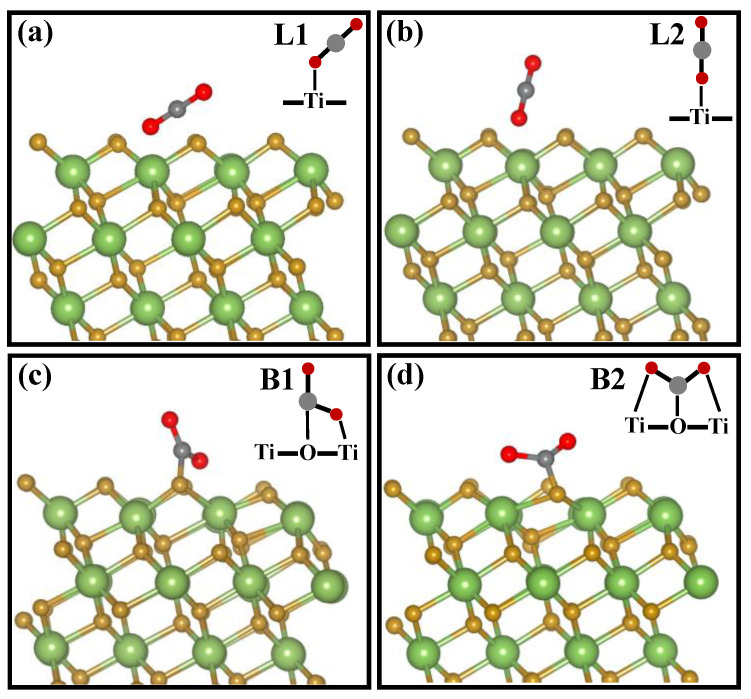
Optimized CO_2_ adsorption geometries on both a pure-phase anatase (112) surface and a (112)_A_/(100)_II_ heterostructure: (**a**) tilted linear; (**b**) perpendicular linear; (**c**) bidentate carbonate; (**d**) bridged carbonate. The inserted images show the corresponding CO_2_ adsorption configurations.

The adsorption of CO_2_ on the catalyst surface is the initial and most vital step in the CO_2_ reduction reaction [7]. The CO_2_ adsorption configurations have a significant influence on the selectivity of catalytic reactions in the following steps [33,34]. Therefore, our study started by investigating the adsorption behaviors of CO_2_ on the anatase surface. In this simulation, four possible configurations for the CO_2_ adsorption were explored and the corresponding optimized geometries are shown in Figure 9. According to the calculated adsorption energies ($E_{ad}=E\left(TiO_{2}/CO_{2}\right)-E\left(TiO_{2}\right)-E(CO_{2})$) (see Table 1), it is found that in all adsorption configurations, the *E*_ad,A/II_ values are more positive than *E*_ad,A_, indicating a weaker adsorption capacity of CO_2_ adsorbed on the (112)_A_/(100)_II_ heterostructure compared to that on the pure-phase anatase surface. This may be attributed to the fact that the formation of a (112)_A_/(100)_II_ heterostructure can induce the charge transfer between anatase (112) and II (100) surfaces. The charge redistribution may break the original stability of the anatase (112) surface and thus cause a slight local deformation on the anatase surface, which is unfavorable for CO_2_ adsorption on the (112)_A_/(100)_II_ heterostructure. In addition, the most stable adsorption configuration of CO_2_ on both pure-phase anatase and the (112)_A_/(100)_II_ heterojunction is a nearly linear configuration (L1), slightly tilted relative to the surface normal through the interaction of the O atom in CO_2_ with surface five-fold-coordinated Ti_5c_ (*d*(Ti_5c_O) = 2.62 and 2.71 Å, respectively) and the C atom in CO_2_ with surface-bridging O_2c_ (*d*(O_2c_C) = 2.70 and 2.78 Å, respectively). The metastable structure also displays a linear configuration (L2), in which the CO_2_ molecule is nearly vertically adsorbed on the top of surface Ti_5c_ atoms through relatively weak interactions with an adsorption energy of −0.02 and 0.21 eV, respectively. In bidentate carbonate (B1, one side of CO_2_ laying along the Ti_5c_–O_2c_ bond) and bridged carbonate (B2, two O atoms in CO_2_ bridging two surface Ti_5c_ and the C atom in CO_2_ pointing downward) adsorption configurations, the CO_2_ molecules are largely deformed with respect to the linear shape and exhibit bent structures. Both of these configurations are unstable and the energy of the B2 structure is even higher than that of B1 by 0.53 and 0.30 eV, respectively. The stability order of the adsorption structures, L1 > L2 > B1 > B2, is similar to the CO_2_ adsorption on anatase (101) surfaces [35,36,37]. Meanwhile, on anatase (001) [33] and rutile (001) surfaces [38], the most stable CO_2_ adsorption structure displays a bent configuration, suggesting that the CO_2_ adsorption behavior is related to both the adsorbed crystal phases and the crystal facets. Based on the above analyses, the L1 configuration is chosen for the following mechanism study of CO_2_ photoreduction on both pure-phase anatase and a (112)_A_/(100)_II_ heterostructure.

**Scheme 1:**
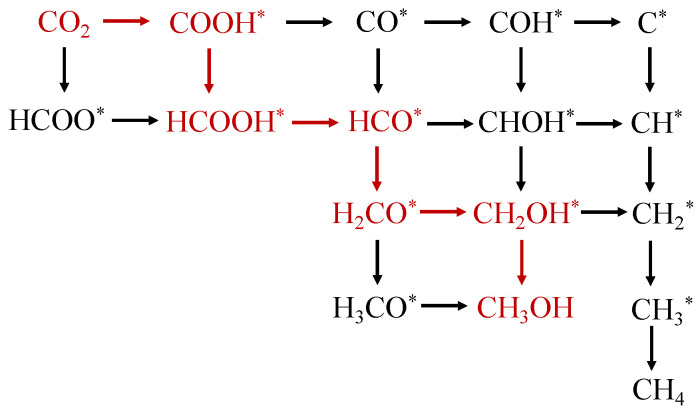
Possible reaction pathways and intermediates of the CO_2_ reduction reaction. The asterisk (*) indicates that the intermediate is adsorbed on the active site. The red parts correspond to the optimal reaction pathways.

**Figure 10:**
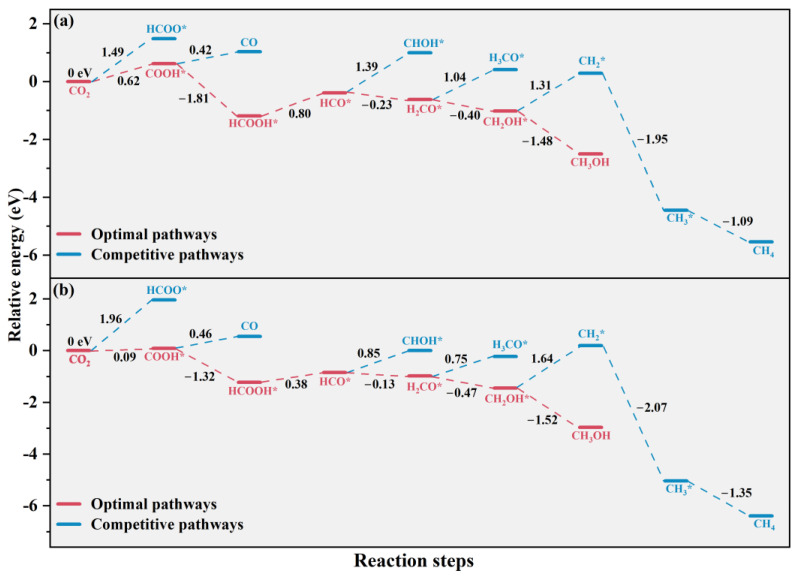
Potential-energy-change profiles of various possible reaction pathways for photocatalytic CO_2_ reduction on (**a**) pure-phase anatase and (**b**) the (112)_A_/(100)_II_ heterostructure. The sum of energies of the CO_2_ molecule and anatase TiO_2_ is the zero reference for energy. The asterisk (*) indicates that the intermediate is adsorbed on the active site.

Possible reaction pathways and intermediates of CO_2_ reduction are listed in Scheme 1 and the potential-energy-change profiles of various possible reaction pathways are displayed in Figure 10. Our investigations show that for the first hydrogenation step on pure-phase anatase, the reaction energy of the formation of COOH* by connecting the H atom to the O atom of CO_2_ is 0.62 eV, which is more thermodynamically favorable by 0.87 eV than the generation of HCOO* through the H atom’s addition to the C atom (see Figure 10a). This is mainly because due to the higher electronegativity of the O atom, as the C and O atoms of CO_2_ are positively and negatively charged, respectively. Hence, the O atom of CO_2_ is more easily hydrogenated to COOH*. COOH* subsequently undergoes hydrogenation to HCOOH or dehydroxylation to CO. The acquisition of HCOOH is an exothermic reaction with an energy decrease of −1.81 eV, whereas the transformation to CO is an endothermic process (0.42 eV). Therefore, CO_2_ is more likely to undergo a two-step hydrogenation mechanism to HCOOH. In addition, the adsorption energy for HCOOH and CO are −1.57 and −0.33 eV, respectively. This suggests that the interaction between HCOOH and anatase is stronger than that of CO, which further confirms that HCOOH is the preferential intermediate product. Then, HCOOH* is hydrogenated and dehydrated to form HCO*, and this step is considered to be the rate-limiting step with the highest energy barrier of 0.80 eV. The next step of HCO* hydrogenation contains two possible pathways—one to H_2_CO* and the other one to CHOH*. The production of H_2_CO* has an exothermic reaction energy of −0.23 eV, while the formation of CHOH* is an endothermic process with a distinct energy increase of 1.39 eV, indicating that the generation of H_2_CO* is more favorable than CHOH*. Subsequently, H_2_CO is more easily reduced to CH_2_OH* intermediate (−0.40 eV), rather than undergoing hydrogenation to form CH_3_O* (1.04 eV). In the following step, there are considerable differences in the reaction energies for the formation of CH_3_OH (−1.48 eV) and CH_2_* (1.31 eV). The significantly negative energy barrier implies that CH_2_OH*’s reduction to CH_3_OH is more competitive relative to CH_2_*. In addition, it is noted that CH_2_OH* → CH_2_* is the rate-limiting step for CH_4_ formation, and its corresponding energy barrier (1.31 eV) is much higher than the rate-limiting energy for CH_3_OH generation (HCOOH* → HCO* with 0.80 eV). Therefore, the final product is more likely to be CH_3_OH. In brief, the optimal route of CO_2_ reduction on pure-phase anatase is CO_2_ → COOH* → HCOOH* → HCO* → H_2_CO* → CH_2_OH* → CH_3_OH, which is similar to the reaction mechanism of CO_2_ reduction on g-C_3_N_4_-based photocatalysts [39,40].

Figure 10b presents the potential energy diagram of CO_2_ reduction reaction pathways on the (112)_A_/(100)_II_ heterostructure. It can be seen that the reaction pathways of CO_2_’s transformation to CO, CH_3_OH, and CH_4_ on the (112)_A_/(100)_II_ heterostructure are the same as those on pure-phase anatase, but the corresponding reaction energy for each step is quite different. On the (112)_A_/(100)_II_ heterostructure, the calculated reaction energy of the initial step of CO_2_’s hydrogenation to the COOH* intermediate is 0.09 eV, which is much smaller than that on pure-phase anatase, at 0.62 eV, indicating that the (112)_A_/(100)_II_ heterostructure is more conductive to the formation of COOH* through a barrierless pathway. This is mainly because when anatase (112) and II (100) surfaces are in contact, there will be charge transfer between the two phases. The charge rearrangement may induce more negative charges around the O atoms of CO_2_ on the (112)_A_/(100)_II_ heterostructure (−0.19 and −0.27 *e*) compared to those on pure-phase anatase (−0.14 and −0.20 *e*). Consequently, the H atom is preferably bound to the O atom of CO_2_ on the (112)_A_/(100)_II_ heterostructure to generate the COOH* intermediate. In addition, although the rate-limiting step of generating CH_3_OH on the (112)_A_/(100)_II_ heterostructure is also the hydrogenation and dehydration of HCOOH* to HCO*, it possesses a lower energy barrier than the pure-phase anatase by 0.42 eV. Moreover, the reaction energy of CH_2_OH*’s reduction to CH_2_* on the (112)_A_/(100)_II_ heterostructure is 1.64 eV, much higher than that on pure-phase anatase (1.31 eV), which suggests that the selectivity of CO_2_ reduction to form CH_3_OH might be enhanced on mixed-phase TiO_2_. In summary, the (112)_A_/(100)_II_ heterostructure is more favorable for the generation of CH_3_OH compared to pure-phase anatase. It is known that commercial P25 is also a mixed-phase TiO_2_. However, many experimental studies have shown that the P25 photocatalyst exhibited excellent photoreduction activity in reducing CO_2_ into CH_4_ [41,42,43], which is different from the main product generated by our constructed anatase/rutile heterostructure model. This distinction may originate from the differences in the exposed surface for the CO_2_ reaction process, proceeding on the anatase (112) surface in our model and the anatase (101) surface in P25 [41,42,43].

## 3. Methods

### 3.1. Calculations of Band Edges

In the heterojunction model, the band information that we could directly obtain was the valence band maximum (VBM) and the conduction band minimum (CBM) positions of the overall system. Then, the valence band offset (VBO) and conduction band offset (CBO) at the mixed-phase junctions were calculated to evaluate how the remaining two band edges were shifted relative to the VBM and CBM. Based on the viewpoint proposed by Ceder et al. that the difference between energy level and Hartree potential remains unchanged everywhere in space [44], the valence band offset value ($E_{VBO}$) can be computed as

$$E_{VBO}=\left({HOMO}_{i}^{bulk}-V_{i}^{bulk}\right)-\left({HOMO}_{ii}^{bulk}-V_{ii}^{bulk}\right)+(V_{i}^{int}-V_{ii}^{int})$$

where i and ii stand for the semiconductors of phase i and phase ii, respectively. ${HOMO}_{sc}^{bulk}$ is the highest occupied molecular orbital (HOMO) energy of the bulk semiconductor. $V_{sc}^{bulk}$ is the electrostatic potential of the bulk semiconductor. Under the periodic boundary condition (PBC), the $V_{sc}^{bulk}$ value is zero. $V_{sc}^{int}$ is the averaged electrostatic potential in the bulk-like region of the heterojunction interface for a semiconductor, which is obtained by nanosmoothing the original electrostatic potential profile with the MACROAVE code [45]. $E_{VBO}>0$ suggests that the photogenerated holes tend to flow from phase ii to phase i.

In the same way, the conduction band offset value ($E_{CBO}$) is given by

(3) $$E_{CBO}=\left({LUMO}_{i}^{bulk}-V_{i}^{bulk}\right)-\left({LUMO}_{ii}^{bulk}-V_{ii}^{bulk}\right)+(V_{i}^{int}-V_{ii}^{int})$$

where ${LUMO}_{sc}^{bulk}$ is the lowest unoccupied molecular orbital (LUMO) energy of the bulk semiconductor. $E_{CBO}>0$ indicates that the photogenerated electrons are likely to transfer from phase i to phase ii.

### 3.2. Computational Setup

All density functional theory (DFT) simulations were performed using the freely available program package CP2K/Quickstep [46]. The Perdew–Burke–Ernzerhof (PBE) functional [47] was employed for ab initio calculations. The basis sets for valence electrons were double-ζ basis functions with one set of polarization functions (DZVP) [48]. The core electrons were represented using analytic Goedecker–Teter–Hutter (GTH) pseudopotentials [49]. The dispersion correction of the Grimme method D3 [50] was adopted to describe the van der Waals interactions. For geometry optimizations, the wave function optimization was carried out using an orbital transformation minimizer, which can give the optimal convergence control [51]. Meanwhile, for absorption spectra calculations, the matrix diagonalization method was applied to obtain the wave function information of unoccupied molecular orbitals for data analysis. The convergence criterion for wave function optimization was set at a maximum electronic gradient of 3 × 10^−7^ a.u. and the plane wave cutoff for the electron density expansion was 400 Ry. It is known that when employing the pure generalized gradient approximation (GGA) functional to estimate the solid electronic structures, there is a delocalization error leading to an underestimation of the band gap. Therefore, to more accurately describe the alignment of electronic energy levels across the heterojunction interface, the band edge positions were computed using the Heyd–Scuseria–Ernzerhof (HSE06) screened hybrid functional [52,53] based on the structures from the PBE functional calculations.

## 4. Conclusions

In this work, the band edges of three-phase junctions in the anatase/rutile mixed-phase TiO_2_ were successfully determined and the mechanism of charge separation and transport across the anatase/rutile interface was revealed based on ab initio simulations. Our results show that the VBM positions decrease in the order of rutile > II > anatase, facilitating the holes’ transfer from anatase to rutile phases under sunlight irradiation. Meanwhile, the CBM position of anatase is slightly higher than that of intermediate II phase, and thus the II will act as an electron trapping site and impede the electrons’ flow from rutile to anatase. However, the potential drop across the anatase/rutile interface is so large that it can provide a relatively high driving force for the photoelectrons’ migration to anatase via the very thin layer of II. Moreover, the work function of II is higher than that of anatase, leading to the formation of a built-in electric field and an upward band bending from anatase to II, which is also favorable for the electrons’ accumulation on the anatase phase. Briefly, electron transfer from rutile to anatase is energetically permitted, as indicated experimentally [17,31]. In addition, it is found that after the construction of the (112)_A_/(100)_II_ heterostructure, its optical absorption capacity is significantly enhanced compared to the pure-phase anatase. The effective utilization of solar light is conductive to promoting the photocatalytic activity.

Simultaneously, the CO_2_ photoreduction mechanism on an anatase (112) surface was systematically investigated. It is confirmed that the most stable CO_2_ adsorption configuration is a nearly linear configuration (L1), slightly tilted with respect to the surface normal, which is similar to the adsorption behavior on an anatase (101) surface [35]. The calculated potential energies suggest that on both pure-phase anatase and the (112)_A_/(100)_II_ heterostructure, CH_3_OH is more liable to be the final product and the corresponding optimal route is CO_2_ → COOH* → HCOOH* → HCO* → H_2_CO* → CH_2_OH* → CH_3_OH. During the whole reaction process, the rate-limiting step is the hydrogenation and dehydration of HCOOH to HCO*. The rate-limiting energy on the (112)_A_/(100)_II_ heterostructure is obviously lower than that on pure-phase anatase, and the first hydrogenation step of CO_2_ to COOH* on the (112)_A_/(100)_II_ heterostructure is thermodynamically spontaneous with a barrierless pathway, demonstrating that anatase/rutile mixed-phase TiO_2_ is an excellent photocatalyst for CO_2_ reduction.

## Figures and Tables

**Table 1:** Calculated adsorption energies (eV) of CO_2_ on the pure-phase anatase (*E*_ad,A_) and the (112)_A_/(100)_II_ heterostructure (*E*_ad,A/II_).

	Adsorption Configuration	*E*_ad,A_	*E*_ad,A/II_
L1	tilted linear	−0.41	−0.39
L2	perpendicular linear	−0.02	0.21
B1	bidentate carbonate	0.34	0.72
B2	bridged carbonate	0.87	1.02

## Data Availability

The data presented in this study are available in this article and the Supplementary Materials.

## Supplementary Materials

The following supporting information can be downloaded at: https://www.mdpi.com/article/10.3390/molecules29174105/s1, Table S1: The average electrostatic potentials of the anatase phase ($V_{anatase}$, eV), vacuum phase ($V_{vac}$, eV), and the electrostatic potential difference (∆V, eV) for the anatase (112) surface; Table S2: The average electrostatic potentials of the rutile phase ($V_{rutile}$, eV), vacuum phase ($V_{vac}$, eV), and the electrostatic potential difference (∆V, eV) for the rutile (101) surface; Table S3: The average electrostatic potentials of the anatase phase ($V_{anatase}$, eV), intermediate II phase ($V_{II}$, eV), and the electrostatic potential difference (∆V, eV) for the (112)_A_/(100)_II_ heterojunction; Table S4: The average electrostatic potentials of the intermediate II phase ($V_{II}$, eV), rutile phase ($V_{rutile}$, eV), and the electrostatic potential difference (∆V, eV) for the (001)_II_/(101)_R_ heterojunction.
