# Supplementary material for: Theoretical Study on Photocatalytic Reduction of CO2 on Anatase/Rutile Mixed-Phase TiO2
Source: Molecules. 2024 Aug 29;29(17):4105. doi: 10.3390/molecules29174105 (PMC11397365; doi:10.3390/molecules29174105)
Supplement: Supplementary file 1 [file molecules-29-04105-s001.zip › molecules-3144983-supplementary.pdf]

Supplementary Information

Theoretical Study on Photocatalytic Reduction of CO_2 on Anatase/Rutile Mixed-Phase TiO_2

Jieqiong Li [1,2], Shiyu Wei [1], Ying Dong [1], Yongya Zhang [1,*] and Li Wang [2,*]

[1] Henan Engineering Center of New Energy Battery Materials, College of Chemistry and Chemical Engineering, Shangqiu Normal University, Shangqiu 476000, China; lijqchem@foxmail.com (J.L.); weishiyu1015@126.com (S.W.); dongying0928@126.com (Y.D.)

[2] Henan Key Laboratory of Protection and Safety Energy Storage of Light Metal Materials, Henan University, Kaifeng 475004, China

* Correspondence: zhangyongya_1990@126.com (Y.Z.); chemwangl@henu.edu.cn (L.W.)

Table S1. The average electrostatic potentials of anatase phase ($V_{anatase}$, eV), vacuum phase (V_{vac}, eV) and the electrostatic potential difference (ΔV, eV) for anatase (112) surface.

Layer	V_{anatas}	V_{vac}	ΔV
4L	-9.42	4.03	13.45
5L	-8.79	4.61	13.40
6L	-8.19	5.18	13.37
7L	-7.72	5.67	13.39
8L	-7.32	6.06	13.38

Table S2. The average electrostatic potentials of rutile phase (V_{rutile}, eV), vacuum phase (V_{vac}, eV) and the electrostatic potential difference (ΔV, eV) for rutile (101) surface.

Layer	V_{rutile}	V_{vac}	ΔV
4L	-9.85	4.01	13.86
5L	-9.07	4.74	13.81
6L	-7.81	5.92	13.73
7L	-7.86	5.85	13.71
8L	-7.46	6.26	13.72

Table S3. The average electrostatic potentials of anatase phase ($V_{anatase}$, eV), intermediate II

phase (V_{II}, eV) and the electrostatic potential difference (ΔV, eV) for $(112)_A/(100)_{II}$ heterojunction.

**Layer**	$V_{anatase}$	V_{II}	ΔV
2L	-0.04	-0.19	0.15
4L	0.06	-0.43	0.49
6L	0.18	-0.46	0.64
8L	0.24	-0.44	0.68

Table S4. The average electrostatic potentials of intermediate II phase (V_{II}, eV), rutile phase (V_{rutile}, eV) and the electrostatic potential difference (ΔV, eV) for $(001)_{II}/(101)_R$ heterojunction.

**Layer**	V_{II}	V_{rutile}	ΔV
2L	-0.17	0.18	0.35
4L	-0.06	0.16	0.22
6L	-0.01	0.08	0.09
8L	0.04	0.10	0.06
